# The Bergen 4-Day Treatment for Panic Disorder: A Pilot Study

**DOI:** 10.3389/fpsyg.2018.01044

**Published:** 2018-06-26

**Authors:** Bjarne Hansen, Gerd Kvale, Kristen Hagen, Kay M. Hjelle, Stian Solem, Beate Bø, Lars-Göran Öst

**Affiliations:** ^1^OCD-Team, Haukeland University Hospital, Bergen, Norway; ^2^Department of Clinical Psychology, University of Bergen, Bergen, Norway; ^3^Psychiatric Department, Hospital of Molde, Molde, Norway; ^4^Department of Psychology, Norwegian University of Science and Technology, Trondheim, Norway; ^5^Kronstad DPS, Haukeland University Hospital, Bergen, Norway; ^6^Department of Psychology, Stockholm University, Stockholm, Sweden

**Keywords:** panic disorder, intensive treatment, exposure, outcome, agoraphobia

## Abstract

The current article reports on the findings from a pilot treatment study on panic disorder (PD) with or without agoraphobia. Consecutively referred patients were included and treated with the Bergen 4-day treatment format. Twenty-nine patients were included, primarily from unsuccessful treatment courses in the Norwegian specialist mental health care system, either ongoing or previously. Prior to treatment, only 34% were able to work but at 3-month follow-up 93% were able to do so. The proportion achieving reliable change on the panic severity measure was 76% post-treatment and 90% at follow-up. The remission rate was 72% at both assessments. These effects are significantly higher than those reported for six standard CBT studies in the literature using the same primary outcome measure (Panic Disorder Severity Scale). It is concluded that the Bergen 4-day treatment is a promising treatment approach for PD, and a randomized controlled trial is warranted.

## Introduction

In outpatient specialist mental health care (SMHC) patients with anxiety and depression represent the largest group and have the longest treatment courses (Whiteford et al., 2013). This rather grim reality exists even though there are evidence-based treatments for all these disorders. The personal and socio-economical gains for each patient that can be helped could be immense given that around 75% of the patients with an anxiety disorder develop the disorder before their mid-20s (Kessler et al., 2005), and that the disorders are typically associated with poor quality of life and daily functioning (Whiteford et al., 2013).

The OCD-Team at Haukeland University Hospital has developed a concentrated exposure treatment (The Bergen 4-day treatment) for obsessive-compulsive disorder (OCD). The treatment is delivered during four consecutive days in groups of 3–6 patients with the same number of therapists. The results show that 83% of the patients are reliably improved and 68% remitted at 6-month follow-up, with very low declining rate and practically no dropout (Havnen et al., 2014, 2017). A recent 4-year follow-up found that 69% of the patients were recovered 4 years after treatment (Hansen et al., 2018).

The 4-day format is planned to be tested in randomized controlled trials for patients with the most common anxiety disorders, namely panic disorder (PD), social anxiety disorder (SAD), generalized anxiety disorder (GAD), depression, and body dysmorphic disorder (BDD). However, the RCTs will only be initiated if diagnosis-specific pilot studies indicate that the clinical effects from the 4-day format are as good as (non-inferior to) the effects of previously published effectiveness studies on evidence-based treatments for the disorder in question (Chambless and Hollon, 1998). Analogous to the approach for OCD, these pilot studies will be conducted as part of the outpatient psychiatric service.

The Bergen 4-day treatment is based on a cognitive behavior therapy approach with a special focus on individually tailored and therapist-assisted exposures. It can best be described as individual treatment in a group setting because it is delivered in groups where the therapist-patient ratio is 1:1. The first of the 4 days is dedicated to explaining the treatment and planning of exposures, and the last to summarizing “lessons learnt” and planning how to ensure that changes becomes an integrated part of normal life (3 h). The two middle days are spent on therapist-assisted exposures interspaced with brief meetings with the group. In these meetings each patient share their experiences of the exposure training and gets feedback on how to deal with challenges. During the 2 days with exposures, patients are taught how to actively approach whatever elicits relevant anxiety or discomfort without simultaneously employing subtle avoidance strategies but rather “lean into the anxiety” (labeled the LET-technique). During these two middle days the patients spend approximately 8 h with the group/therapist and then practice on their own in the evening.

The present pilot study on PD describes clinical changes at post-treatment and 3-months follow-up and patient satisfaction with the treatment, and the results presented are part of the quality assessment procedure, which is an integrated part of the 4-day treatment. The results are also compared with previously published studies of CBT that have used the Panic Disorder Severity Scale (PDSS) as the primary outcome measure. The main aims of the present study were to investigate the feasibility, acceptability, and treatment outcome of the 4-day treatment for PD. Based on our experience of the 4-day treatment for OCD as described above, we expect that this treatment will be as effective as standard CBT using weekly sessions.

## Materials and Methods

### Patients

#### Referral Procedures and Diagnostics

The patients were referred to the specialist health care by their individual general practitioner, and if their symptoms were severe enough they were offered treatment as a part of public health care. In the period October 2016 to April 2017, patients at Kronstad psychiatric clinic (Bergen, Norway) who fulfilled the diagnostic criteria of PD with or without agoraphobia were offered participation in the 4-day treatment. Time-slots for 4-day groups were decided in advance. Clinicians at Kronstad were informed about the 4-day treatment and were encouraged to refer patients with ongoing but unsuccessful treatments. This means that patients were offered participation successively upon availability in groups. Group treatment would not be offered if the patient was suicidal, psychotic, actively abusing alcohol or narcotics, bipolar in an active phase, or did not speak Norwegian. Twenty-nine patients were given a diagnosis of PD according to Diagnostic and statistical manual for mental disorders, 5^th^ ed. (DSM-5) criteria (American Psychiatric Association, 2013). Of these 29 participants 21 had agoraphobia with PD, whereas eight had PD without agoraphobia.

#### Background Information

**Table 1** displays a summary of background data for the sample. There was a majority of female patients (69%) and the mean age of the patients was 31.9 years (range: 18–53) with a self-reported duration of PD being 5.9 years.

**Table 1:** Background information on the patients (*N* = 29).

Variable	*M* (*SD*)	*N* (%)
Gender: females		20 (69.0)
Mean age	31.9 (11.1)	
Duration of the disorder (years)	5.9 (6.4)	
Currently in ongoing treatment		18 (62.1)
Duration of current Tx (months)	11.7 (8.7)	
Previous treatments		
None		6 (20.7)
One		14 (48.3)
Two or more		9 (31.0)
Marital status		
Single		9 (31.0)
Married or cohabiting		19 (65.5)
Unknown		1 (3.4)
Educational status		
Junior high school		5 (17.2)
High school		15 (51.7)
College		7 (24.1)
Unknown		2 (6.9)
Work status		
Working		9 (31.0)
Unable to work		19 (65.5)
Unclear		1 (3.4)
Time out of work (months)	13.9 (15.0)	
Comorbidity		
Yes		16 (55.2)
No		13 (44.8)
Psychotropic medication		
SSRIs		8 (27.6)
Bensodiazepines		6 (20.7)

At the time of inclusion 18 (62.1%) of the patients were in active treatment courses without successful results, with an average duration of 11.7 months (range: 2–30). In total, 23 (79.3%) of the patients had previous treatment courses for the PD; 14 patients (48.3%) had obtained one treatment course, six patients (20.7%) had two, one patient (3.4%) had three, and two patients (6.9%) had received five treatment courses. Of these, three reported earlier exposure treatment, three reported Internet-based cognitive behavior therapy, and 17 had received what was described as psychotherapy, medications, or other forms of therapy. The mean scores on PDSS for patients with or without earlier treatment did not differ significantly, *t*(27) = 0.31, *p* = 0.76.

With respect to marital status, 14 (48.3%) of the patients were in a relationship, five (17.2%) were married, nine (31.0%) were single, and one unknown. Regarding education, five (17.2%) had finished junior high school, 15 (51.7%) had finished high school, seven (24.1%) had a higher education (university or similar), and two were unknown.

Before treatment, 19 (65.5%) of the patients were unable to work due to their PD. For these patients the average time out of the job market was 13.9 months.

Comorbidity included major depressive disorder (5) hypochondriasis/health anxiety (2), generalized anxiety disorder (4), social phobia (1), insomnia (1), post-traumatic stress disorder (1), atypical anorexia (1). Twelve of the included patients had no current comorbidity. The mean PDSS scores before treatment for patients without comorbidity was not significantly different from patients with comorbidity, *t*(27) = 0.62, *p* = 0.54.

A total of 13 patients reported use of psychotropic medications. Eight patients had been on stable doses of Selective Serotonin Reuptake Inhibitors (SSRI; five on Sertraline, three on Escitalopram) prior to treatment, and three patients were on stable doses of sleeping medicine (two on Zopiclone and one Alimemazine). Four patients were on antipsychotic medication (three with Quetiapine and one on Levomepromazin). Six patients had prescriptions of benzodiazepines to be taken when needed. No changes were enforced with respect to medication, but the patients were informed that the use of anxiety reducing medication (benzodiazepines) was prohibited during the 4-day treatment.

### Assessment

Patients answered standardized self-report questionnaires online at pre-treatment and post-treatment. The questionnaires covered symptoms of PD, agoraphobia, generalized anxiety, and depression. If patients did not complete self-report questionnaires according to a pre-set time limit, an automatic text message was sent to their phones. An independent assessor conducted a standardized phone interview using the PDSS (see below) at pre- and post-treatment and follow-up.

#### Diagnostic Interview

Before treatment started, the patients went through a diagnostic interview by a trained psychologist using the Structured Clinical Interview for DSM-5 (First et al., 2015). Comorbid disorders were evaluated using the Mini International Neuropsychiatric Interview (MINI; Sheehan et al., 1998) by an experienced therapist. MINI is a short structured diagnostic interview which screens axis-1 DSM-IV disorders, and the Norwegian version has good psychometric properties (Mordal et al., 2010).

#### Panic Disorder Severity

The PDSS (Shear et al., 1997) which consists of seven items, is a validated interview for assessing PD severity. The PDSS was administered via a standardized phone interview by a clinician trained in the interview. Internal consistency was good to excellent in the present study with Cronbach’s α of 0.76 at pre-treatment, 0.88 at post-treatment and 0.89 at follow-up. PDSS has been shown to be sensitive to change in PD severity (Shear et al., 1997). Along with the diagnostic interview and PDDS, the assessor rated patients on the Clinical Global Impressions Scale (Severity and Improvement) (CGI; Guy, 1976). The CGI provides an overall summary scored on a 1–7 scale which takes into account all available patient information.

#### Depressive Symptoms

Symptoms were measured with the Patient Health Questionnaire 9 (PHQ-9; Kroenke et al., 2010). PHQ-9 is a nine item questionnaire for measuring severity of depressive symptoms, and total scores range from 0 to 27.

#### Symptoms of Generalized Anxiety

It was measured with the Generalized Anxiety Disorder (GAD-7; Spitzer et al., 2006). GAD-7 is a seven item questionnaire which has been proven to be a good measure of generalized anxiety, with excellent psychometric properties. Total scores range from 0 to 21.

#### Treatment Satisfaction

In order to obtain a measure of the patients’ degree of satisfaction with the 4-day treatment the Client Satisfaction Questionnaire (CSQ-8; Larsen et al., 1979) was applied at post-treatment assessment. This is an eight-item questionnaire that measures patient satisfaction with health services, where the items are rated from 1 (very low satisfaction) to 4 (very high satisfaction). The total score ranges from 8 to 32, with higher scores indicating higher degree of satisfaction. The CSQ-8 has good psychometric properties, with high internal consistency (Cronbach’s α = 0.93), and high inter-item correlation (Larsen et al., 1979).

#### Work Status and Ability to Work After Treatment

To measure patients’ ability to work, they were asked by the assessment team about work status at post-treatment and at 3-month follow-up. The patients were also asked about whether their anxiety symptom hinders their ability to work. This information was combined to form a dichotomous variable consisting of participants that were able to work vs. unable to work.

### Procedure

Before treatment was initiated a diagnostic screening that combines information from patient’s self-report questionnaires with diagnostic interviews was conducted. If the patient fulfilled the diagnostic criteria for PD and there was group availability a video explaining the 4-day treatment was shown^1^, and subsequently the therapist elaborated and answered any questions the patient may have. The video underscored that in order for the treatment to be helpful the patient had to make an active decision to get rid of the disorder and to fully engage in the treatment. At the end of the screening an adapted version of the Borkovec and Nau (1972) *Reaction to treatment scale* was used to assess treatment expectations of outcome and treatment credibility. The patient was asked to evaluate four aspects of expectancy and credibility on a 0–100% scale, and if a number below 70% was reported, it served as an opportunity to clear up misunderstandings. If the patient wanted to initiate treatment, he/she would be granted place in a treatment group, and would watch a second video^2^ which elaborated the content of the treatment, the structure, and the focus of the 4 days. It was underscored that these 4 days will be fully dedicated to the treatment, and that the patients were required to have no other appointments. This also applied to the evenings of days 2 and 3. Patients also received written information about the group, and were informed that they should come up with exposure tasks to be used during the treatment. One week prior to treatment, the leader of the group would call each patient to make sure they had received all necessary information and were ready to begin treatment.

### Treatment

The patients were given the 4-day treatment based upon a manual for PD originally developed for the treatment of OCD and adapted for treatment of PD. The treatment was delivered during four consecutive days, and also included a session (with no exposure) 3 months after treatment. The treatment was delivered in groups of 3–6 patients, with a therapist-patient ratio of 1:1. There was also a psycho-educative meeting for relatives or close friends on the third day.

The main focus of the 4-day treatment is the “LEaning in Technique” (LET), which emphasizes a shift from avoiding unpleasant symptoms, thoughts and emotions, to instead actively approach whatever elicits relevant anxiety/discomfort and “lean into the anxiety.”

The first day, all patients and therapists met for 3–4 h. A thorough psychoeducation covering core characteristics of PD, maintaining factors, treatment principles and exposure technique were presented. All patients also prepared individual exposure tasks for days 2 and 3. The second and third days (each approximately 8 h) were allocated to individually tailored and therapist-assisted exposure in as many relevant settings as possible, interspaced with brief meetings for all patients and therapists. Examples of subtle avoidance were demonstrated, and each patient was assisted to practice the LET technique on their most relevant exposure tasks. During the two exposure days, all patients together with their therapist approached numerous anxiety-provoking situations, as they practiced the LET-technique. They were encouraged to practice in as many relevant situations as possible, as each situation is considered an opportunity to create change. In the case of exposure for a PD, leaning into the anxiety may involve physical exercises and hyperventilation to increase physical arousal, in addition to a mental attempt to overload your heart and “cause a heart attack,” instead of avoidance and down-regulation of anxiety.

Also the patients practiced to identify exactly when they were tempted to initiate subtle avoidance, and the patients were taught that these are the moments with the largest potential for change, since this is where they actively can choose to do something incompatible (“leaning in”) with having the disorder. If they had difficulties doing this, it was suggested to “fake it until you make it.”

Typically, in a group of 3–6 patients, not all “get the essence” of the LET-technique at the same time, and the group meetings in the morning, lunch, and late afternoon at the 2 days of exposure training served to help the ones lagging behind to dare to “hunt for anxiety without holding back.” At these meetings each patient reported on their progression, and at the end of each report, the patients rated their own performance on a scale from 1 to 6, where 6 indicated that they did “not hold back” during the exposures. Since the score referred to behavior that they were able to control (“Leaning in” or “doing something incompatible with having the disorder” vs. “holding back”) it was within reach for everyone to obtain a score of 6. The group leader asked each patient focused questions of whether they were able to find anxiety (“was the task relevant for eliciting anxiety”) and whether they were (“holding back,” “leaning in,” or “doing something incompatible with the disorder”). If they were holding back, this was typically followed by questions about how to manage to “lean in” on the next exposure tasks. At some point in time the exposure tasks that used to elicit anxiety might no longer be potent, which might be seen as one of the main goals of the treatment. But even in these situations, the reduction of anxiety was not given much attention. Rather the focus was on whether there is any “gold (anxiety eliciting situations or thoughts) to be found elsewhere – “we do not want to leave any behind,” and on the LET-technique. By using this approach all patients were able to rate their own LET-performance with a rating of 6 at the end of the second day of exposure therapy.

Exposure plans for each evening were made, and each patient reported back to the therapist by text messages to inform whether the agreed upon exposure tasks were completed. On the last day each patient made an individual exposure plan for the next 3 weeks in order to take responsibility for their own recovery and make the changes an integrated part of their lives, without assistance from the clinic. On the last day the principles of change and how to conduct exposures were repeated and important principles to maintain robust and enduring change were discussed. The patients reported their use of the LET technique daily on-line the first 3 weeks following treatment, but with no feedback from the therapists.

### Therapists

All treatment groups were led by 4-day experts, with extensive experience with the protocol (treatment format and rationale) and treatment of anxiety disorders. Certification of a group leader involves participation of a minimum of six groups. In order to be certified as a 4-day therapist one must have earlier experience in exposure-based treatment of anxiety disorders, and participate in a minimum of two treatment groups. All therapists involved in the present groups were licensed clinical psychologists, psychiatrists or last-year clinical psychology students, who were certified 4-day treatment therapists.

### Data Completion

A total of 29 patients are included in the dataset. Of the 29 study participants, 28 were available for PDSS-interview post-treatment, and 27 (93.1) at 3-month follow-up. A total of 25 (86.2%) patients completed self-report questionnaires at post-assessment.

### Definition of Response and Remission

Because there are no international consensus criteria for PD, as there are for OCD (Mataix-Cols et al., 2016), we decided to use three different criteria that have been applied previously in evaluating the clinical outcome for anxiety disorders.

#### The Jacobson and Truax (1991) Criteria

First, the patient’s change score from pre-treatment to post-treatment (or follow-up) on the PDSS must be statistically reliable (larger than the scale’s measurement error) at the 0.05-level [Reliable Change Index (RCI)]. Second, the post-treatment (follow-up) score should more probably belong to the distribution of the normal population than that of the patient population. RCI was calculated using the present sample’s pre-treatment SD and the internal consistency reported by the scale constructors (Shear et al., 1997) yielding a RCI of seven points. The cut-off score was calculated as the patient sample’s pre-treatment mean minus 2^∗^SD; 15.79–2(3.79) = 7.85, i.e., a score of 7. Thus, using the Jacobson and Truax criteria treatment response is defined as a change of at least seven points. Remission was defined as a post-treatment score of seven or less and a change of at least seven points.

#### The Furukawa et al. (2009) Criteria

Response was defined as a reduction of the pre-treatment PDSS score of at least 40%. A patient was considered remitted if the post-treatment score fell in the “borderline ill” category or better, which is 5 for PD and 7 for PD with agoraphobia.

#### The Otto et al. (2016) Criteria

The Clinical Global Impressions-Severity Scale (CGI-S) was employed to determine remission status. The remission criterion was fulfilled if the patient had a post-treatment score on CGI-S of 1 (normal) or 2 (borderline mentally ill) and had no panic attacks. In this study we used item 1 from PDSS to evaluate the presence of panic attacks. If a patient had 0 or 1 on this item this part of the criterion was considered fulfilled.

### Statistical Analyses

There were few incidents of missing data. Only 3.5% of data were missing on the PDSS across the three assessment times (one at post-treatment and two at follow-up). Missing data were replaced using the expectation-maximization method of SPSS, version 24. This method was chosen to allow for repeated measures ANOVA. Statistical analyses were conducted with all 29 patients included. Repeated measures ANOVA for PDSS, GAD-7, and PHQ-9 were conducted using Greenhouse–Geisser corrections when Mauchly’s test of sphericity was significant. To calculate effect sizes of change over time Cohen’s *d* was used, defined as (*M* pre-treatment – *M* post-treatment)/*SD* pre-treatment (Morris and DeShon, 2002). Comparison of the present results with those of studies evaluating standard CBT for PD using the PDSS as primary outcome measure was done with independent *t*-test for continuous variables and Fisher’s exact probability test for categorical variables.

## Results

### Clinical Outcome

As expected the patients showed a significant and large reduction in PD symptoms (measured with PDSS) from pre- to post-treatment, and the change remained stable 3 months after treatment (see **Table 2**). Mauchly’s test was significant (*p* = 0.044), therefore Greenhouse–Geisser correction was applied. The repeated measures ANOVA indicated significant change in PD symptoms, (1.657) = 89.89, *p* < 0.001, $\eta_{\text{p}}^{2}=0.762$. There was a significant change in severity from pre- to post-treatment (*p* < 0.001), but no significant change from post-treatment to follow-up (*p* = 0.50). For CGI-S there were also significant and large reductions in PD symptoms, Wilk’s Lambda = 0.058, *F*(2,27) = 217.87, *p* < 0.001, $\eta_{\text{p}}^{2}=0.942$. As with PDSS, there was a significant change in CGI-S severity from pre- to post-treatment (*p* < 0.001), and no change from post-treatment to follow-up (*p* = 0.64). There was also a significant reduction in depressive symptoms, Wilk’s Lambda = 0.568, *F*(1,28) = 21.29, *p* < 0.001, $\eta_{\text{p}}^{2}=0.432$, and for symptoms of generalized anxiety, Wilk’s Lambda = 0.296, *F*(1,28) = 66.476, *p* < 0.001, $\eta_{\text{p}}^{2}=0.704$ from pre- to post-treatment.

**Table 2:** Results (M and SD) on the primary and secondary outcome measures.

				Cohen’s *d*	
Variable	Pre	Post	F-up	Pre–post	Pre-f-up
PDSS	15.79 (3.97)	5.34 (4.22)	4.82 (3.65)	2.63	2.76
CGI-S	5.03 (0.57)	0.97 (0.87)	1.07 (1.13)	7.12	6.95
GAD-7	11.93 (3.76)	6.13 (3.06)		1.54	
PHQ-9	10.79 (5.93)	6.72 (4.48)		0.69	

*PDDS, Panic Disorder Severity Scale; GAD-7, Generalized Anxiety Disorder-7; PHQ-9, Patient Health Questionnaire-9. d = Cohen’s d (M_pre_ – M_post_)/SD_pre_.*

### Response and Remission

**Table 3** displays the treatment response and remission rates based on three different criteria. For the Jacobson and the Furukawa criteria PDSS was used whereas CGI-S was utilized for the Otto criteria. All three criteria yielded very high response proportions; 76–97% at post and 82–97% at follow-up. The Jacobson RCI on the other hand requires at least a seven-point reduction irrespective of the pre-treatment PDSS score. It is surprising that the stringent CGI-S criterion (≥50% reduction) yielded the highest proportion of responders, which might be due to the construction of this single item measure. The remission rates were also high but varied less than the response rates; 72–79% post-treatment and 72–83% at follow-up.

**Table 3:** Response and remission rates at post and follow-up using different criteria (*N* = 29).

	Jacobson and Truax, 1991	Furukawa et al., 2009	Otto et al., 2016
*Response*	Seven points reduction	40% reduction	Three points reduction on CGI-S^∗^
Post	75.9%	82.8%	96.6%
Follow-up	89.7%	96.6%	82.8%
*Remission*	≤7 on PDSS	≤5 or 7 on PDSS	1 or 2 on CGI-S and no PA
Post	72.4%	79.3%	72.4%
Follow-up	72.4%	72.4%	82.8%

*^∗^We decided on this criterion, which is ≥50% reduction, since Otto et al. (2016) did not have a response criterion in their study. CGI-S, Clinical Global Impressions-Severity Scale; PDSS, Panic Disorder Severity Scale; PAs, panic attacks.*

**Table 4** displays the clinical improvement at post-treatment and follow-up using the Jacobson and Truax (1991) criteria since they were the most conservative of those in **Table 3**. None of the patients had significantly deteriorated at post-treatment or at follow-up. Both at post-treatment and follow-up, 21 of the 29 patients (72.4%) were remitted. Looking at the post-treatment remitters, 16 of the 21 (76%) patients retained their status at follow-up, while five (24%) showed some worsening and fell in the responder category. The single patient with treatment response status post was remitted at follow-up, whereas four of the seven unchanged (57%) patients had remitted, and the remaining three were still unchanged at follow-up.

**Table 4:** Clinical improvement at post and at 3-month follow-up according to the Jacobson and Truax criteria.

	Status at follow-up				
Status at post-treatment	Remission	Response	No change	Deterioration	Total
Remission	16	5	0	0	21 (72.4%)
Response	1	0	0	0	1 (3.5%)
No change	4	0	3	0	7 (24.1%)
Deterioration	0	0	0	0	0
Total	21 (72.4%)	5 (17.3%)	3 (10.3%)	0	29

### Work Ability

There was an increase in the patients’ ability to work from pre-treatment to follow-up. Before treatment, 19 (65.5%) of the patients were too impaired by their PD to be working (on sick leave), and 9 (32.1%) patients were able to work. Three months after treatment 25 of 27 patients (92.5%) were able to work, while two (7.5%) were not. This change was significant as tested with McNemar’s test (*p* < 0.001).

### Treatment Satisfaction

All patients reported high satisfaction with the 4-day treatment. Patients said that the treatment had helped them deal more effectively with their problems, and 93.1% described that the treatment had met “almost all” or “most of my needs.” All patients would definitely recommend the treatment to a friend in need, 100% were “very satisfied” or “mostly satisfied” with the amount of help they got, and 82.8% of the patients rated the quality of the service as “excellent,” and all patients reported that they received the kind of service they wanted. Overall, the patients reported high satisfaction with the 4-day treatment. A summary of the CSQ-8 scores is displayed in **Table 5**.

**Table 5:** Post-treatment scores on Client Satisfaction Questionnaire-8.

		Scale points			
Item	Topic	1	2	3	4
1	Quality of service	0	0	5	24
2	Kind of service	0	0	7	22
3	Met needs	0	2	6	21
4	Recommend to a friend	0	0	0	29
5	Amount of help	2	0	4	23
6	Deal with problems	0	0	4	25
7	Overall satisfaction	0	0	5	24
8	Come back	1	0	3	25

*Mean CSQ-8 score was 30.16 (2.68), range is 23–32, median = 31 (possible range is 8–32). N = 29.*

### Benchmarking: Comparison With Standard CBT

In order to obtain a perspective on the outcome of this pilot study we searched the PsycINFO database for CBT studies of PD with or without agoraphobia, which had used PDSS as the primary outcome measure. We found nine such studies (Barlow et al., 2000; Nakano et al., 2008; Bergström et al., 2010; Nordin et al., 2010; Wims et al., 2010; Nations et al., 2012; Beutel et al., 2013; Meyerbroeker et al., 2013; Otto et al., 2016) with 517 patients in total. There were six on standard face-to-face CBT with weekly sessions and three on Internet-based CBT with weekly modules. The average treatment period in the face-to-face treatments was 10 weeks (range: 5–13) with a total treatment time of 14.4 h (range: 6.5–24), and a mean intensity (hours of therapy per week) of 1.5 (range: 0.8–2.0). The corresponding figures for our pilot study are 4 days and 23 h. Comparisons on background variables showed that there was no significant difference regarding mean age; pilot group 31.9 years and standard CBT 35.0 years, *t*(517) = 1.53, *p* = 0.17, or mean duration; 5.9 and 7.6 years respectively, *t*(381) = 1.02, *p* = 0.31. Proportion of female patients (69 vs. 64%) and proportion taking prescribed psychotropic medication (48.3 vs. 51.6%) for the pilot group and standard CBT did not differ significantly. Proportion of patients diagnosed with agoraphobia did not differ between the pilot group (75.9%) and standard CBT (87.3%), but regarding comorbidity the pilot group (55.2%) had a significantly higher proportion (Fisher’s exact probability test, two-tailed, *p* = 0.013), than standard CBT (31.1%). However, the 4-day treatment had zero percent attrition whereas the standard CBT conditions had 18%, a significant difference (Fisher’s test, two-tailed, *p* = 0.009). The follow-up assessment was performed after 3 months in the 4-day treatment and on average after 4.1 months (range: 1–6) in standard CBT.

**Table 6** presents the comparison on PDSS and at pre-treatment the pilot group had a significantly higher mean than the combined standard CBT from the literature. However, at post-treatment the pilot group had a significantly lower mean, whereas there was no difference at follow-up. The mean pre–post change scores were 10.45 versus 7.36, and pre-follow-up 10.97 versus 9.51, for the pilot group and standard CBT, respectively, but these differences cannot be tested without the individual patients’ values.

**Table 6:** Comparison between the Bergen 4-day treatment and standard CBT on PDSS.

	*Bergen 4-day*		*Standard CBT*		*Statistic*
*Time point*	*N*	*M (SD)*	*N*	*M (SD)*	*t*-Test (*p* =)
Pre	29	15.79 (3.97)	276	14.39 (3.48)	2.03 (0.043)
Post	29	5.34 (4.22)	262	7.03 (3.95)	2.17 (0.031)
Follow-up	29	4.82 (3.65)	255	4.88 (4.00)	0.08 (0.939)

*Data for Standard CBT were collected from the following studies: Bergström et al. (2010); Nordin et al. (2010), Wims et al. (2010); Nations et al. (2012), Beutel et al. (2013), and Otto et al. (2016), which have data on remission (**Table 7**).*

**Table 7** displays the comparisons on remission rate using the three sets of criteria described earlier. These showed that the 4-day treatment yielded significantly higher remission rates than standard CBT, irrespective of which criterion used. At post-treatment 72–79% of patients in the 4-day format were remitted versus 51% for standard CBT, and at follow-up the numbers were 72–83% for intensive treatment versus 52% for standard treatment.

**Table 7:** Comparison between the Bergen 4-day treatment and standard CBT on proportion of remission.

	Criteria set			
	Jacobson	Furukawa	Otto	Standard CBT
Post	72.4^a^	79.3^b^	72.4^a^	51.3
Follow-up	72.4^a^	72.4^a^	82.8^b^	51.8

*Data for Standard CBT were taken from the published studies using the PDSS as the primary outcome measure: standard CBT used four different criteria of remission: (1) free from PD-diagnosis, (2) CGI-Severity rating of 1 or 2, (3) CGI-Improvement rating of ≤2, and (4) PDSS score of ≤5 for PD, ≤7 for PDA. Superscript letters indicate difference in relation to standard CBT. ^a^p < 0.05; ^b^ p < 0.01.*

## Discussion

This is the first pilot study where the Bergen 4-day treatment is given to patients with PD. Nearly 80% of the patients in the current pilot study had previous unsuccessful treatment courses, while at the time of referral 62% was receiving ongoing unsuccessful treatment courses with an average length of 12 months in the specialist health care, and were referred by their therapists due to lack of improvement. The aim of this pilot study was to test the feasibility, acceptability, and clinical outcome of the Bergen 4-day treatment for PD with or without agoraphobia.

The clinical outcome was also very good with a change score on the PDSS equivalent to an effect size of 2.63 at post and 2.76 at 3-month follow-up. On the CGI-Severity scale the within-group effect size was extremely large; 7.12 at post and 6.95 at follow-up. Finally, using the most conservative criteria, the proportion of responders was 75% post-treatment and 90% at follow-up, whereas the proportion in remission was 72% both at post-treatment and follow-up assessment.

In the group of patients 66% were unable to work due to their PD and they had been out of the job market for 14 months on average. After the treatment 93% reported that they were working or able to work. This dramatic change in ability to work after only 4 days of treatment could represent an immense gain in quality of life, personal, and socio-economical functioning.

Furthermore, the patients’ satisfaction with the received treatment was very high with a mean post-treatment CSQ-8 score of 30.2 when the maximum is 32. The patients were highly satisfied with the 4-day treatment, regarding amount, quality, and relevance of the treatment. Furthermore, all patients would recommend the treatment to a friend who might need treatment for PD, and all patients said that would return to the clinic if they needed.

Moreover, 55% had various comorbid disorders and 48% were taking psychotropic drugs for their PD. When comparing this pilot sample with the combined patient sample from nine studies of standard CBT there were no significant differences regarding mean age, duration of PD, proportion of females, proportion with agoraphobia, or proportion on prescribed psychotropic drugs. However, the pilot group had a significantly higher proportion with comorbid disorders. Thus, it can be concluded that the present sample was at least as severe, if not more so, as the combined standard CBT sample from published studies.

When comparing the treatment effect on PDSS we found that the pilot group had a significantly higher pre-treatment and a significantly lower post-treatment score than the mean of six standard CBT-studies, whereas the follow-up difference was non-significant. Also, the proportion achieving remission was significantly higher for the pilot group both at post-treatment and at follow-up. Significant changes were also seen in measures of depression and generalized anxiety. A tentative conclusion is that the 4-day treatment yielded at least as good treatment effects as standard CBT carried out over 10 weeks. However, it should be emphasized that these kinds of statistical comparisons are open to questioning since the samples may differ on some variables which have been found to predict outcome, but which have not been described in the published studies, which were published 2010 to 2016. Based on these results, the initiation of a RCT to test the effectiveness for the Bergen 4-day treatment of PD is recommended.

## Ethics Statement

This paper uses data collected as part of the standard assessment procedure at the outpatient OCD-clinic in Helse Bergen, as approved by the Data Protection Official, August 5, 2012.

## Author Contributions

BH, GK, and L-GÖ contributed to the study design. BH, GK, KMH, BB, and KH contributed to data collection. SS, KH, KMH, and L-GÖ conducted the data analyses. All authors contributed to interpretation of the data, drafting of the manuscript, and approved the final version.

## Conflict of Interest Statement

The authors declare that the research was conducted in the absence of any commercial or financial relationships that could be construed as a potential conflict of interest.

## Footnotes

1 https://youtu.be/nqx8knpy3i4

2 https://youtu.be/1Fnxt0_ljpY
